# Infrared Spectroscopy of RNA Nucleosides in a Wide Range of Temperatures

**DOI:** 10.3390/life14040436

**Published:** 2024-03-25

**Authors:** Susana Iglesias-Groth, Franco Cataldo, Martina Marin-Dobrincic

**Affiliations:** 1 Instituto de Astrofisica de Canarias, Via Lactea s/n, 38200 La Laguna, Spain; 2 Actinium Chemical Research Institute, Via Casilina 1626A, 00133 Rome, Italy; franco.cataldo@fastwebnet.it; 3 Applied Physics and Naval Technology Department, Universidad Politécnica de Cartagena, C/Doctor Fleming, s/n., 30202 Cartagena, Spain; martina.marin@edu.upct.es

**Keywords:** astrobiology, astrochemistry, spectral line, identification, methods, laboratory, molecular, molecular data

## Abstract

The RNA world hypothesis suggests that early cellular ancestors relied solely on RNA molecules for both genetic information storage and cellular functions. RNA, composed of four nucleosides—adenosine, guanosine, cytidine, and uridine—forms the basis of this theory. These nucleosides consist of purine nucleobases, adenine and guanine, and pyrimidine nucleobases, cytosine and uracil, bonded to ribose sugar. Notably, carbonaceous chondrite meteorites have revealed the presence of these bases and sugar, hinting at the potential existence of nucleosides in space. This study aims to present the infrared spectra of four RNA nucleosides commonly found in terrestrial biochemistry, facilitating their detection in space, especially in astrobiological and astrochemical contexts. Laboratory measurements involved obtaining mid- and far-IR spectra at three temperatures (−180 °C, room temperature, and +180 °C), followed by calculating molar extinction coefficients (*ε*) and integrated molar absorptivities (*ψ*) for corresponding bands. These spectral data, along with *ε* and *ψ* values, serve to provide quantitative insights into the presence and relative abundance of nucleosides in space and aid in their detection.

## 1. Introduction

**Scheme 1:**
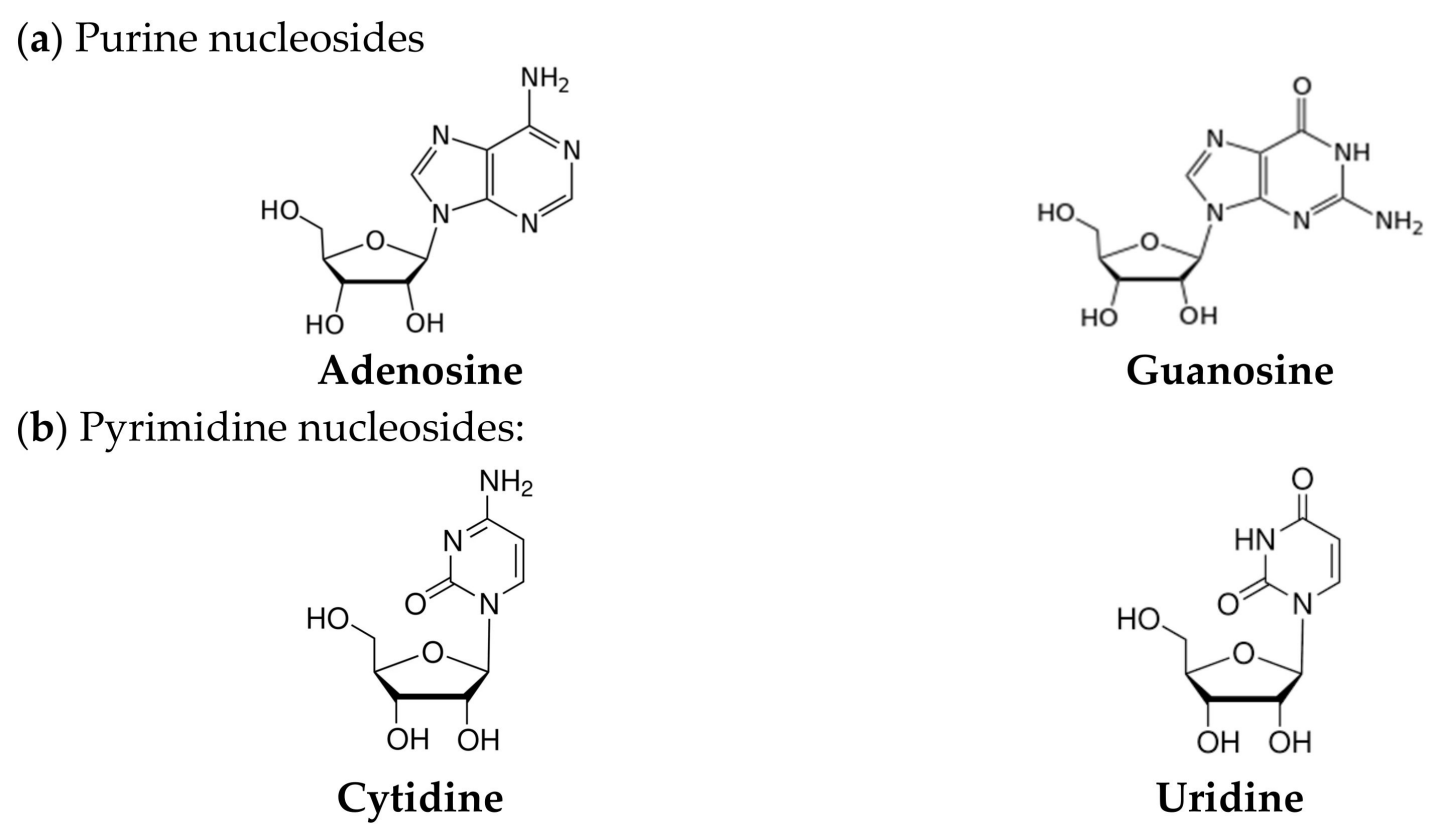
(**a**) Purine nucleosides: adenosine and guanosine (**b**) Pyrimidine nucleosides: cytidine and uridine.

Nucleosides represent a step ahead in molecular complexity with respect to the nucleobases. When the purine and pyrimidine nucleobases are linked through the β-glycosidic bond to ribose or deoxyribose sugars, the resulting RNA or DNA nucleosides are obtained [1]. Based on contemporary life theory, the fundamental principle revolves around an RNA molecule’s ability to self-replicate independently, disseminating essential life functions’ information, thereby driving the quest for its nucleosides in space. The present study is focusing on the infrared spectroscopy of the RNA nucleosides whose chemical structures are shown in Scheme 1.

According to the RNA world theory, in the pre-history of the biochemical evolution of life, RNA has played a key role both as genetic information storage for early life stages but also as a biochemical catalyst [2]. In other words, there was a stage in the pre-history of terrestrial biochemistry where primitive ancestors of modern cells were based exclusively on RNA for the storage of genetic information but also for the normal functioning of the cell, including the RNA replication [3]. Only in a later stage of biochemical evolution was the RNA specialized in the actual roles of information transfer and transduction, while the phenotype passed under the control of proteins through an RNA-peptide world transition stage [2].

The prebiotic synthesis of nucleosides has encountered many more difficulties than the prebiotic synthesis of nucleobases, because of the higher molecular complexity of the former [1]. However, more recent studies discovered potential and compelling prebiotic chemical routes for the abiotic production of nucleosides [4]. The key point regards the fact that the purine and pyrimidine nucleobases can be formed (as can the amino acids) either under prebiotic conditions on the Earth [5,6] but also in conditions occurring in the interstellar medium [7], including chemical processes occurring in star-forming regions [5,8]. Indeed, all the purines and pyrimidines nucleobases were found in carbonaceous chondrites [9], also including some nucleobases not currently used by terrestrial biochemistry. Similarly, sugar ribose was also detected in primitive meteorites [10]. Thus, although the nucleosides were not yet detected in primitive meteorites, it is possible to affirm that the key molecules to produce nucleosides (nucleobases from one side and ribose sugar from the other side) are present in primitive meteorites, and the glycosylation reaction which leads to nucleosides can be induced by cosmic rays [11]. In addition, nucleosides can be produced without the assistance of cosmic rays, but rather through wet chemistry conditions [8,12]. The study of nucleoside radiolysis has demonstrated that adenosine and guanosine, which are purine-based, are more resistant to radiolysis compared to cytidine and uridine, which are pyrimidine-based. This explains why adenine and guanine, the parent purine nucleobases, were more readily detected and found in higher concentrations in carbonaceous chondrites than cytosine and uracil, the parent pyrimidine RNA nucleobases [13,14].

On these premises, it is possible that RNA nucleosides may be detectable in space as is the case with nucleobases. The previous work on infrared spectroscopy was indeed dedicated to the nucleobases [15], while the present work is focusing on the infrared spectroscopy of the RNA nucleosides within our endeavors to explore spectroscopically and molecules of astrobiological relevance, like, for instance, amino acids, potentially detectable in space [16,17]. References to the significance of mid-infrared and far-infrared spectroscopy in studying astrobiologically relevant compounds, as well as the latest telescopes, may be found in the sources [18,19].

## 2. Materials and Methods

### 2.1. Materials

All the RNA nucleosides, i.e., purine-based adenosine (Ado) and guanosine (Guo), as well as the pyrimidine-based cytidine (Cyd) and uridine (Urd) (see Scheme 1 for the chemical structures), used in the present work were purchased from Aldrich-Merck (St. Louis, MO, USA-Darmstadt, Germany). The cesium iodide spectroscopic matrix, which was used to create the pellets, was also acquired from Aldrich-Merck.

### 2.2. Preparation of Laboratory Apparatus and Pellets for Measuring the Integrated Molar Absorptivity in the Mid- and Far-Infrared Regions

Previous works [17,20] provided detailed descriptions of the laboratory equipment used for low and high temperature spectra in the mid- and far-infrared. Moreover, the comprehensive protocol on the manufacture of the pellets used to calculate the molar extinction coefficient (*ε*) and the integrated molar absorptivity (*ψ*) of the nucleobases studied in the present work can be found in [15,16,17]. Cesium iodide was used as a glue to compact the mix of our sample into pellets in order to conduct FT-IR spectroscopy. In these conditions, the sample analyzed can be considered the solute and the solid CsI matrix as the solvent. Therefore, operating the pellet sample is analogous to handling a solid solution. In such conditions, the determination of Epsilon (ε, molar extinction coefficient) and Psi (*ψ*, integrated molar absorptivity) values does not require a calibration curve, but a single measurement at a high dilution is sufficient.

## 3. Results and Discussion

### 3.1. Mid-Infrared Emissions of Ado, Guo, Cyd, and Urd and Their Molar Extinction Coefficients and Integrated Molar Absorptivity

The molar extinction coefficient (or molar attenuation coefficient) can be expressed using the Lambert-Beer law as follows:

*ε* = *A* (*bc*)^−1^ (1)

i.e., for a specific wavenumber $\tilde{\nu }$ = *λ*^−1^ or (wavelength *λ*) in the infrared spectrum, the absorbance *A* is multiplied by the path length b of the same matrix (expressed in cm) and the concentration of the sample (expressed in mol·L^−1^)) to obtain the molar extinction coefficient *ε* (expressed in L· mol^−1^ · cm^−1^) [17]. The integrated molar absorptivity *ψ* is obtained by integrating the absorbance across the full absorption band, with the $\tilde{\nu }$ in wavenumber on the x-axes:

(2) $$\psi =\int_{band}\;\varepsilon d\tilde{\nu }={\left(bc\right)}^{-1}\int_{band}Ad\tilde{\nu }$$

In an infrared spectrum where wavenumbers are plotted on the x-axis, the integrated absorptivity is measured in units of cm^−1^. If the path length, denoted as *b*, is also expressed in centimeters (cm), and the concentration is given in moles per cubic centimeter (mol·cm^−3^), then the dimension of *ψ* is in centimeters per mole (cm·mol^−1^). To convert this into the astrochemical practical value of km·mol^−1^, we can simply multiply by a factor of 10^−5^ [15,16,17].

Table 1, Table 2, Table 3 and Table 4 show the outcomes of our assessment of both *ε* and *ψ* values of the RNA nucleosides adenosine (Ado), guanosine (Guo), cytidine (Cyd), and uridine (Urd) which are the building blocks for the chemical structure of the informational polymer RNA. 

**Figure 1:**
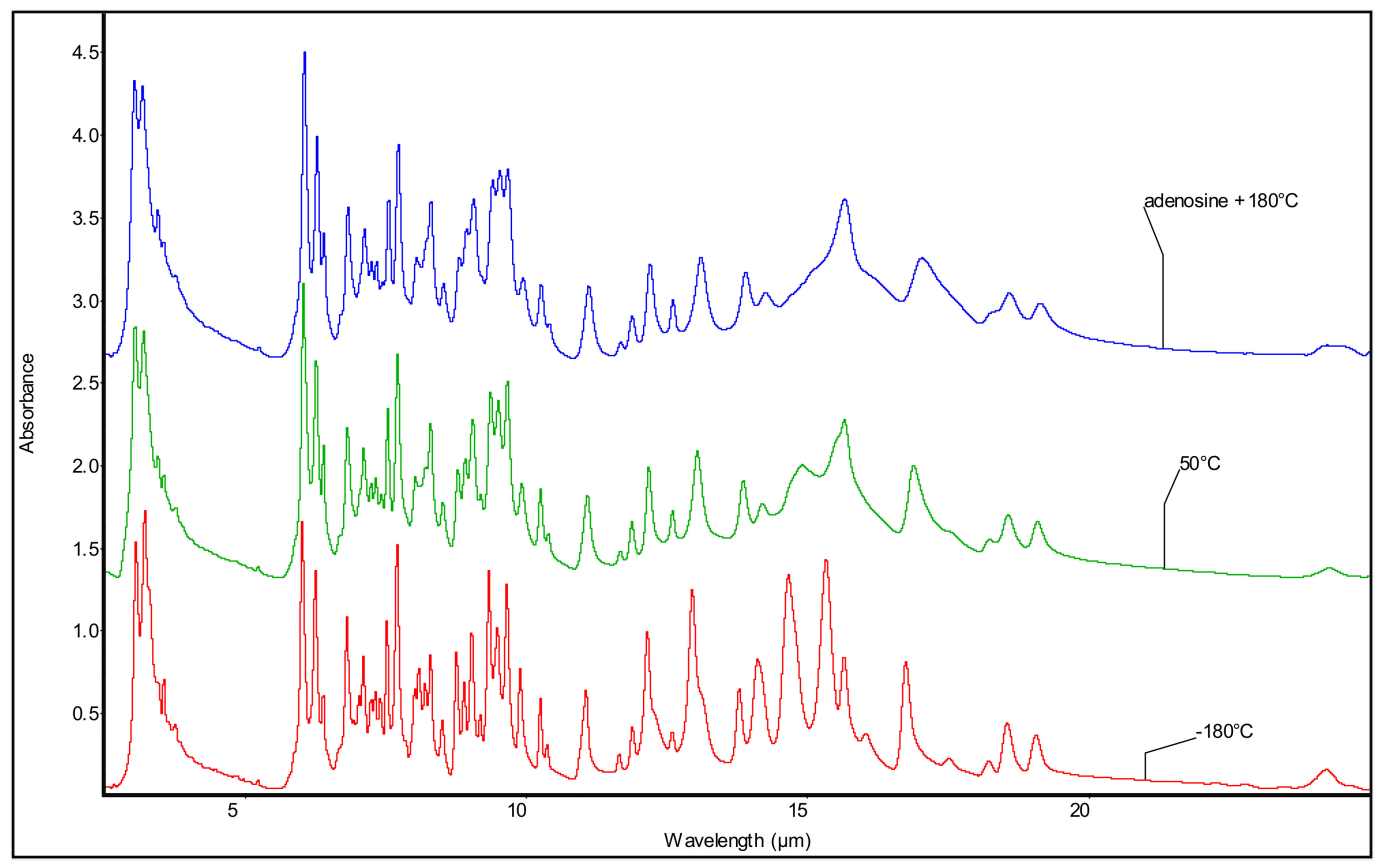
FT-IR spectra of adenosine in CsI matrix recorded (from top to bottom) at +180 °C, +50 °C and −180 °C, respectively.

**Figure 2:**
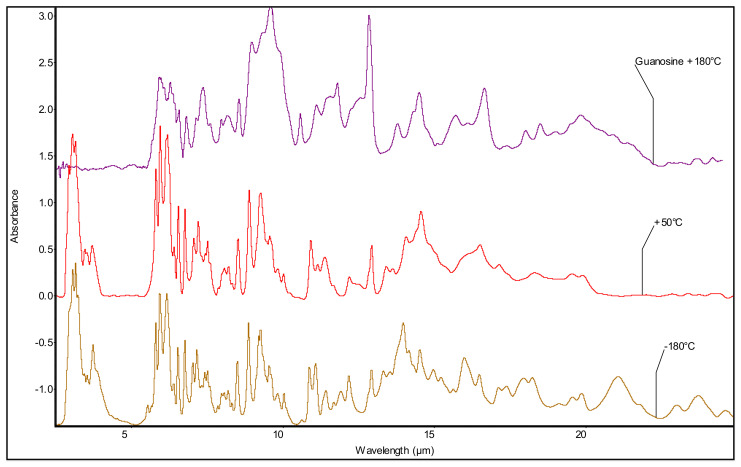
FT-IR spectra of guanosine in CsI matrix recorded (from top to bottom) at +180 °C, +50 °C and −180 °C, respectively.

**Figure 3:**
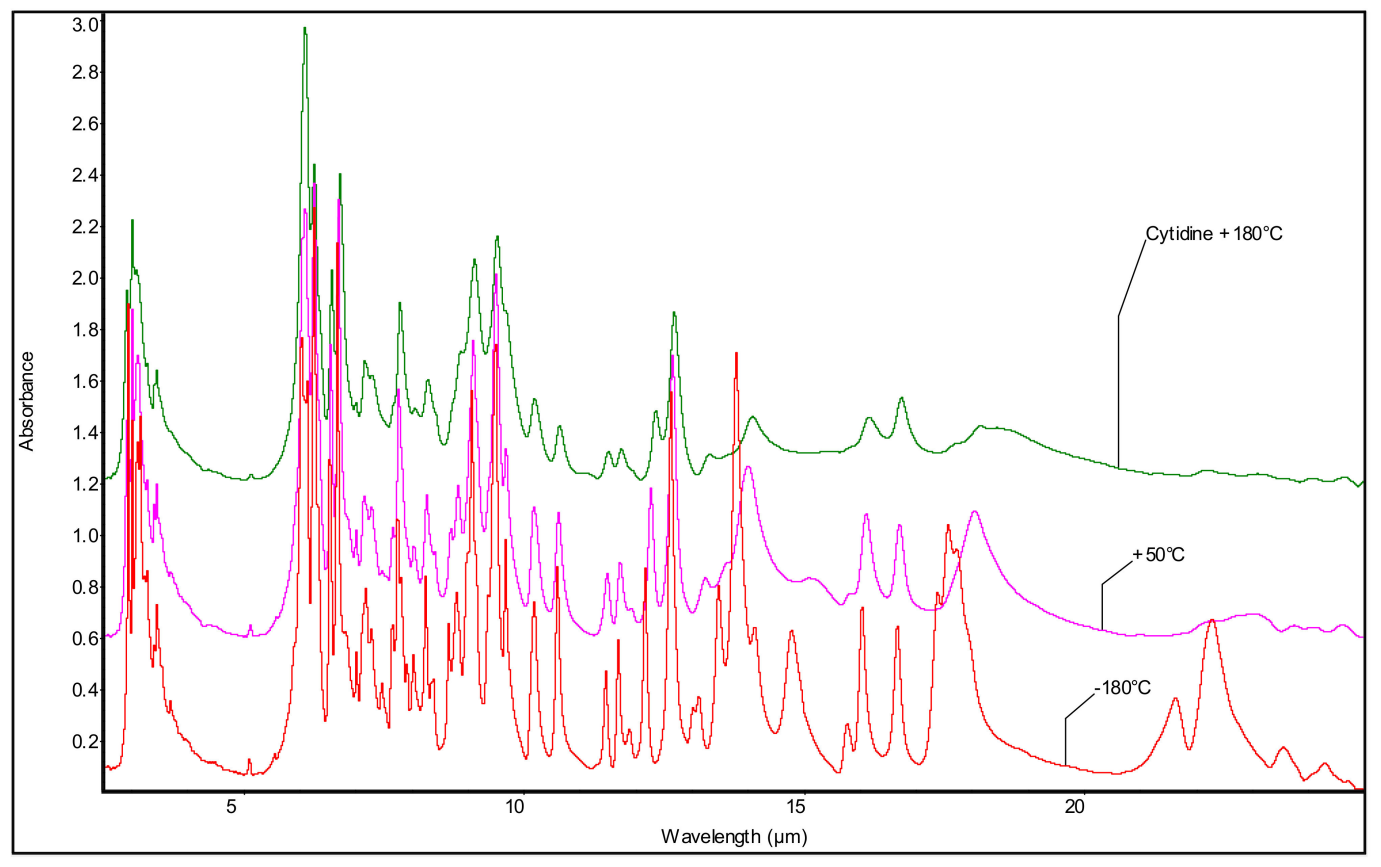
FT-IR spectra of cytidine in CsI matrix recorded (from top to bottom) at +180 °C, +50 °C and −180 °C, respectively.

**Figure 4:**
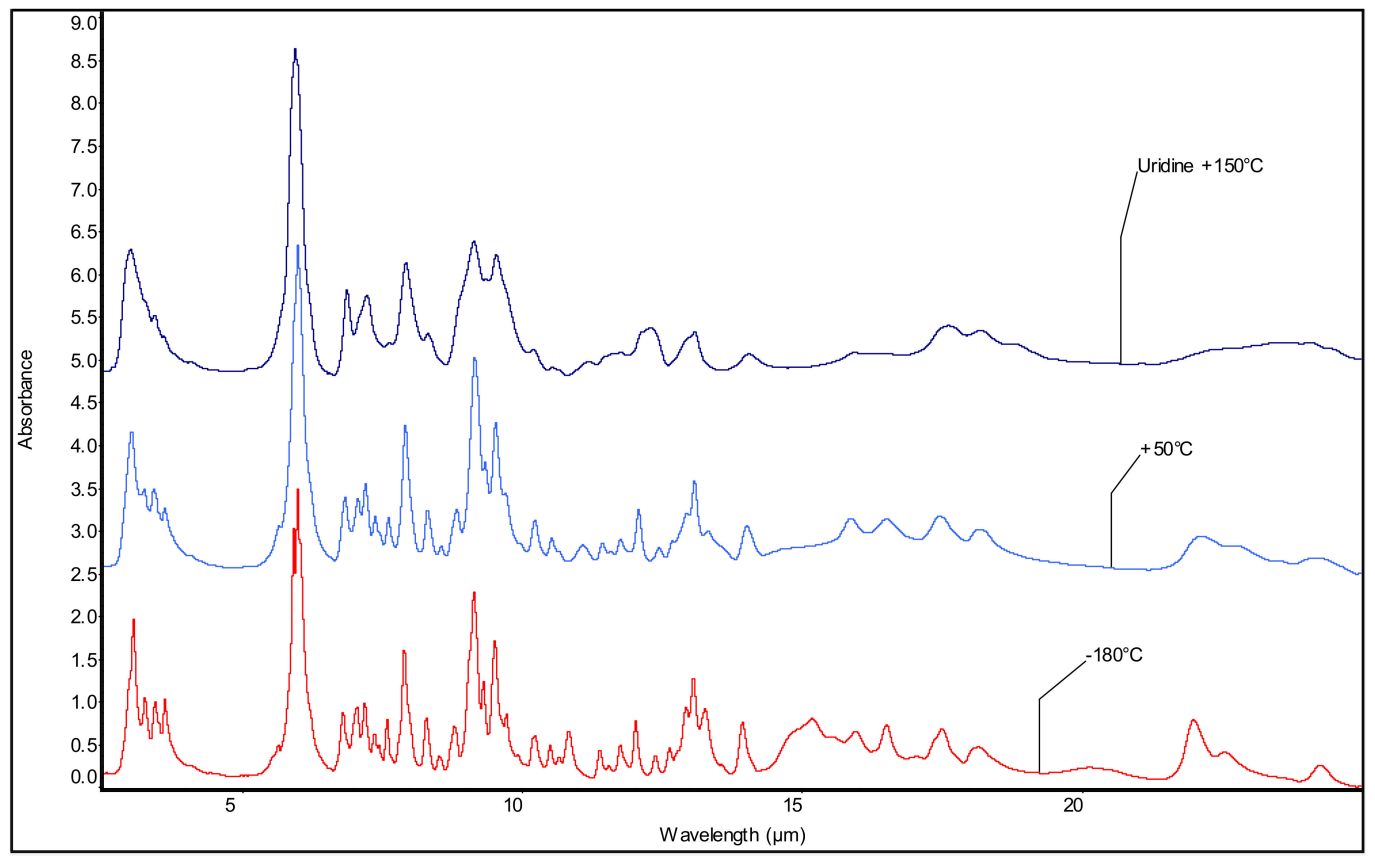
FT-IR spectra of uridine in CsI matrix recorded (from top to bottom) at +150 °C, +50 °C and −180 °C, respectively.

The data are extracted from the spectra illustrated in Figure 1, Figure 2, Figure 3 and Figure 4. Concerning the integrated molar absorptivity, Table 1, Table 2, Table 3 and Table 4 also provide information on the integration ranges within which the *ψ* value for each pertinent band was calculated.

The spectra of the RNA nucleosides are presented with wavelength (μm) on the x-axis, aligning with the format preferred by astrophysicists and consistent with prior studies [15,16,17]. It is important to note that both the determination of band positions and the calculation of integrated molar absorptivity and molar extinction coefficients were conducted using spectra with the x-axis in wavenumbers (cm^−1^) which are the preferred units among spectroscopists. 

As previously mentioned in sections covering amino acids [15,16,17], it is important to emphasize that the molar absorptivities reported were obtained under specific laboratory conditions and should be used with caution when calculating the abundance of nucleobases in space. Although they can provide information about the comparative frequencies of these species when observed in similar spectral regions and allow approximate estimations of abundance levels, accurate determinations will require measuring the absorptivity values of nucleosides under laboratory conditions that closely mimic those found in interstellar space.

### 3.2. Mid-Infrared Spectroscopy of the Purine Nucleosides: Ado and Guo

There are several works on the infrared spectroscopy on the purine nucleosides adenosine (Ado) and guanosine (Guo) (see Scheme 1 for the chemical structures) mainly made with the purpose of bands assignment, base pairing, hydrogen bonding, and as a key step toward the understanding of the infrared spectra of the nucleotides, polynucleotides, and RNA. Without aiming to be comprehensive, we mention a selection of papers dealing with the spectroscopy of purine nucleosides [21,22,23,24,25,26,27,28,29]. The primary objective of this study is to provide the astrophysics and astrochemistry/astrobiology community with the mid-infrared spectra of purine nucleosides (Figure 1 and Figure 2, Table 1 and Table 2) as a means of potentially identifying these compounds in condensed form in space.

Furthermore, the molar extinction coefficients (*ε*) and the integrated molar absorptivity (*ψ*) values (Table 1 and Table 2) were determined for the first time as a tool for the potential quantitative estimation of the amount of (Ado) or (Guo), in case they could be identified in space. Thus, we will not deal with the details of band assignments, already done effectively by many authors, rather, we will focus on some relevant infrared bands which are susceptible to large shifts in the wide temperature range studied. Since the nucleosides are more sensitive to thermal decomposition than the nucleobases [15,30] and the amino acids [16,17], the maximum temperature reached in this study was limited to 180 °C.

In line with earlier research on the spectroscopy of amino acids that are significant to astrobiology, covering a wide range of temperatures [15,16,17], in Table 1, Table 2, Table 3 and Table 4, we have provided a comprehensive analysis of the shift in the mid-infrared band with respect to temperature according to the following:

Δ*ν* = *ν*_(−180 °C)_ − *ν*_(+180 °C)_ (3)

We can see that Δ*ν* denotes the overall displacement of the band, encompassing the complete range of temperatures under consideration. The infrared bands susceptible of large or significant band shift on these molecules is typically assigned to hydrogen bond interaction [15,16,17]. Being a weaker bond than normal covalent bonds, the hydrogen bond is much more sensitive to temperature changes [31]. Consequently, the IR spectral investigation in a wide range of temperatures facilitates identification of the infrared bands involved in hydrogen bonding [32]. 

In Table 1 (see also Figure 1) are reported the infrared band shifts observed in Ado. Of course, the band shifts observed in Ado do not correspond necessarily to the shifts previously measured in the free nucleobase adenine. The reason is certainly due to the presence of the D-ribose ring attached to the purine base which leads to supplementary hydrogen bonding phenomena. At 3.00 μm (3335 cm^−1^), assigned to the OH groups of ribose [22], Δ*ν* = −19 cm^−1^ and more pronounced shift Δ*ν* = −38 cm^−1^ can be observed at 3.16 μm (3167 cm^−1^), assigned to the stretching of the NH_2_ group of Ado [22]. Other important band shifts are observed at 5.20 μm (1923 cm^−1^), with Δ*ν* = 10 cm^−1^ due to combination band, then 6.00 μm (1667 cm^−1^) Δ*ν* = 10 cm^−1^ due to scissoring of the NH_2_ group of Ado, while 6.23 μm (1605 cm^−1^) Δ*ν* = 9 cm^−1^ and 6.63 μm (1508 cm^−1^) Δ*ν* = 9 cm^−1^ are both assigned to the in-plane adenine ring mode [25,26,27]. Other vibrational modes characterized by relatively high band shift and assigned to the ribose ring of the Ado are those at 7.39 μm (1353 cm^−1^) Δ*ν* = 8 cm^−1^, 9.33 μm (1072 cm^−1^) Δ*ν* = 8 cm^−1^, 13.02 μm (795 cm^−1^) Δ*ν* = 9 cm^−1^, and 16.86 μm (593 cm^−1^) Δ*ν* = 10 cm^−1^ [22,25,26,27]. As shown in Figure 1 and Table 1, the maximum temperature reached in the FT-IR spectra recording on Guo was limited to 180 °C, although the melting point of Guo is found at 234°–237 °C. The latter temperature was never reached to avoid any possible thermal decomposition of Guo.

The other purine nucleoside, guanosine (Guo), is more thermally labile than Ado, although its melting point is reported at 239 °C. Figure 2 shows that at 180 °C, the infrared spectrum of Guo is strongly altered, especially at higher frequencies. Consequently, as shown in Table 2, the band shift Δ*ν* from 2.5 μm (4000 cm^−1^) to 5.77 μm (1733 cm^−1^) was determined in the range from −180 °C to +50 °C. Despite these limitations, large band shifts with temperature were measured at 3.12 μm (3209 cm^−1^) Δ*ν* = −9 cm^−1^ and 3.65 μm (2737 cm^−1^) Δ*ν* = −27 cm^−1^ assigned, respectively, to the N-H stretching and to the C-H stretching [23]. Then, apart from the 6.22 μm (1609 cm^−1^) Δν = 12 cm^−1^ due to guanine in-plane mode and the mixed modes due to guanine and ribose overlap both at 7.02 μm (1425 cm^−1^) Δν = 22 cm^−1^ and at 11.35 μm (881 cm^−1^) Δ*ν* = 9 cm^−1^, all the other bands with significantly large band shift reported in Table 2 are assigned to ribose: 7.29 μm (1372 cm^−1^) Δ*ν* = 8 cm^−1^, 8.85 μm (1130 cm^−1^) Δ*ν* = 13 cm^−1^, 9.79 μm (1021 cm^−1^) Δ*ν* = 9 cm^−1^, 11.64 μm (859 cm^−1^) Δ*ν* = 9 cm^−1^, 14.03 μm (713 cm^−1^) Δ*ν* = −11 cm^−1^, and 21.98 μm (455 cm^−1^) Δ*ν* = −9 cm^−1^ [23]. Even the glycosidic bond linking the purine guanine with ribose is subjected to band shift with temperature 8.45 μm (1179 cm^−1^) Δ*ν* = 9 cm^−1^ [23].

### 3.3. The Emissions of the Pyrimidine Nucleosides: Cyd and Urd in Mid-IR

Figure 3 presents the FT-IR spectra of the pyrimidine nucleoside cytidine (Cyd), while Table 3 provides the infrared bands along with their respective *ε* and *ψ* values. Since the melting point of Cyd is found in the range between 210° and 220 °C, the maximum temperature reached for the FT-IR recording was limited to 180 °C to avoid any decomposition which may occur on melting.

As in the case of the purine nucleosides, the infrared spectra in a wide range of temperatures permits to identify the infrared bands directly involved in hydrogen bonding as those which undergo the largest shifts. For Cyd, these bands are located at 2.90 μm (3448 cm^−1^) Δ*ν* = −24 cm^−1^, due to ribose OH stretching [33,34,35]. Other ribose assigned infrared bands which are involved in hydrogen bonding are as follows: 6.08 μm (1646 cm^−1^) Δ*ν* = −8 cm^−1^ ribose ring bending mode, 7.10 μm (1409 cm^−1^) Δ*ν* = 9 cm^−1^, 8.33 μm (1200 cm^−1^) Δ*ν* = 11 cm^−1^, 8.80 μm (1136 cm^−1^) Δ*ν* = 10 cm^−1^, 12.25 μm (816 cm^−1^) Δ*ν* = 12 cm^−1^, 21.65 μm (462 cm^−1^) Δ*ν* = 29 cm^−1^, and 23.75 μm (421 cm^−1^) Δ*ν* = −9 cm^−1^ [23,34,35]. Of course, a series of vibrational modes due to the cytosine moiety of Cyd are also involved in the hydrogen bonding, starting from the bending of the amino group at 6.67 μm (1500 cm^−1^) Δ*ν* = 11 cm^−1^ and followed by a series of bending modes of the pyrimidine ring at 8.67 μm (1153 cm^−1^) Δ*ν* = 9 cm^−1^, 13.61 μm (735 cm^−1^) Δν = −9 cm^−1^, 13.99 μm (715 cm^−1^) Δ*ν* = 15 cm^−1^, 17.39 μm (575 cm^−1^) Δ*ν* = 10 cm^−1^, and 17.73 μm (564 cm^−1^) Δ*ν* = 21 cm^−1^. 

The FT-IR spectrum of the other RNA pyrimidine nucleoside, uridine (Urd) is shown in Figure 4, and the summary of ε and *ψ* parameters can be found in Table 4. Among the RNA nucleosides, the simplest band pattern is displayed by Urd. Since the melting point of Urd is in the range 165°–168 °C, the FT-IR spectra of Urd were recorded from −180 °C to +150 °C.

In the case of Urd, there are only six infrared bands that show a significantly large band shift with temperature and are hence involved in hydrogen bonding. The band at 3.28 μm (3051 cm^−1^) Δ*ν* = −9 cm^−1^ is due to the OH stretching of the ribose moiety [26,27,34,36]. Similarly, the band at 3.47 μm (2878 cm^−1^) Δ*ν* = 9 cm^−1^ is also due to the C-H stretching of the ribose ring, while the band at 11.39 μm (878 cm^−1^) Δ*ν* = −17 cm^−1^ occurs in the bending modes of ribose [36]. There are three other infrared bands with a large shift with temperature in Urd, assigned to the uracil moiety of Urd at 5.95 μm (1681 cm^−1^) Δν = −15 cm^−1^, 7.16 μm (1397 cm^−1^) Δν = 8 cm^−1^, and 12.03 μm (831 cm^−1^) Δν = 8 cm^−1^ [26,27,34,36].

## 4. Conclusions

Astronomers now have access to the reference spectra of the four building blocks of the RNA molecule, adenosine (Ado), guanosine (Guo), cytidine (Cyd), and uridine (Urd), containing the basic information for the initial building of life. The presented spectra can be used to identify nucleosides qualitatively and quantitatively in space. The mid-infrared spectra of the nucleosides were measured at three distinct temperatures: −180 °C, +50 °C, and +180 °C (unless otherwise indicated, refer to Figure 1, Figure 2, Figure 3 and Figure 4). Table 1, Table 2, Table 3 and Table 4 provide a summary of the extent to which the bands shift in function of temperature for all the primary absorption bands. The values for the integrated molar absorptivity (*ψ*) and the molar extinction coefficients (*ε*) of the primary infrared bands of the nucleosides have been calculated and are shown in Table 1, Table 2, Table 3 and Table 4. With the available data, it will be feasible to search for—and potentially identify—the nucleosides and use the *ψ* and *ε* obtained here to assess their local abundance.

## Figures and Tables

**Table 1:** Molar extinction coeff. [*ε*] and integr. molar absorptivity [*ψ*] of ADENOSINE IR emissions.

Wavelength at 50 °C	Wavenumber at 50 °C	Band Shift Δ*ν_a_* −180 °C to +180 °C	Integration Region	[*ε*]	[*ψ*]
μm	cm^−1^	cm^−1^	μm	M^−1^ cm^−1^	km/mol
2.99	3335	−19	3706–2301	320	1404
3.04	* 3289				
3.15	3167	−38	3420–2972	301	447
3.19	3135	3		286	
3.19	* 3127				
3.26	* 3061				
3.38	2958	6		139	
3.40	2933	1		155	
3.42	2919	−3		162	
3.51	2843	2		135	
3.61	2770			92	
3.68	* 2712				
3.73	2676	7		86	
3.80	* 2627				
5.20	1923	10		20	
5.84	1711	3	1776–1523	161	224
5.99	1667	10	1723–1630	409	72
6.01	1664			379	
6.03	1656			263	
6.06	1648			200	
6.23	1605	9	1630–1581	302	33
6.35	1573	5	1581–1558	197	10
6.63	1508	9		52	
6.78	1474	4	1522–1440	201	31
6.90	* 1448				
7.00	1427	6		107	
7.07	1414	4	1440–1396	164	14
7.21	1387	4	1396–1379	123	2,6
7.29	1371	4	1379–1361	133	3,4
7.39	1353	8	1361–1343	102	2,0
7.50	1333	6	1343–1318	233	14
7.68	1302	3	1318–1266	285	32
7.83	* 1277				
7.99	1251	5		125	
8.05	* 1242				
8.17	1224	4		136	
8.27	1209	1	1266–1187	203	40
8.48	1178	3	1187–1157	95	4.3
8.74	1143	7	1157–1134	136	6.0
8.88	1126	5	1134–1119	149	3.2
9.02	1108	5	1119–1097	195	10
9.16	1091	3	1157–1085	102	40
9.32	1072	8		230	
9.47	1055	5		215	
9.63	1038	2	1085–1020	251	55
9.89	1011	5	1020–990	113	5,5
10.22	978	1	988–950	112	7.8
10.37	964	4		56	
11.06	904	4	924–870	102	13
11.64	859	2	870–853	32	0.7
11.84	844	−1	853–835	67	2.9
12.15	823	4	835–806	136	8.7
12.57	795		806–787	82	1.9
13.02	768	9	787–738	151	13
13.10	* 763				
13.83	723	6	735–713	116	4,5
14.16	706	6		88	
14.88	672			133	
15.31	* 653				
15.62	640	1	714–603	187	51
16.00	* 625				
16.86	593	10	603–554	133	12
17.48	* 572				
18.21	549	1		46	
18.55	539		554–496	75	8.8
19.08	524	2		67	
24.27	412		422–405	11	0.7

(*) New band at low T.

**Table 2:** The [*ε*] and [*ψ*] parameters of GUANOSINE IR emissions.

Wavelength at 50 °C	Wavenumber at 50 °C	Band Shift Δ*ν_a_* −180 °C to +180 °C	Integration Region	[*ε*]	[*ψ*]
μm	cm^−1^	cm^−1^	μm	M^−1^ cm^−1^	km/mol
2.80	3570	2 ***		141	
2.84	* 3523				
2.87	* 3483				
2.89	3466	−6 ***		310	
2.93	* 3417			0	
3.00	3333	4 ***	3625–2985	375	1259
3.00	* 3330				
3.01	3318	4 ***		377	
3.02	* 3314				
3.03	* 3299				
3.04	* 3292				
3.05	* 3283				
3.11	3220	3 ***		353	
3.12	* 3210				
3.12	3209	−9 ***		350	
3.18	3143	2 ***		259	
3.19	3130			249	
3.21	* 3111				
3.22	* 3106				
3.37	* 2964				
3.38	2958	−2 ***	3625–2447	99	1806
3.42	2924	−3 ***	2985–2502	120	205
3.45	2896			115	
3.50	2860	2 ***		102	
3.53	2830	7 ***		84	
3.56	* 2809				
3.65	2737	−27 ***		114	
5.13	1950				
5.50	* 1819				
5.61	* 1781				
5.77	1733	3 ***	1784–1713	320	39
5.89	* 1697		1850–1554		434
5.91	1693	7	1713–1663	406	47
6.10	1639			355	
6.15	1625		1663–1591	376	89
6.22	1609	12		298	
6.37	1569		1598–1553	118	7.1
6.50	1538	6	1553–1507	223	34
6.72	1487		1507–1456	225	30
			1455–1289		107
7.02	1425	22	1450–1407	134	10
7.16	1396		1407–1379	177	11
7.11	* 1406				
7.26	1377				
7.29	1372	8			
7.40	1352			97	
7.47	1338		1636–1317	132	11
7.56	1323	4		77	
7.66	* 1305				
7.83	1277			19	
7.94	1259	−2		48	
8.03	1245			63	
8.17	1224	−5	1289–1198	68	27
8.29	1207			30	
8.45	1179	9	1198–1157	128	19
			1157–940		178
8.85	1130	13	1154–1111	247	31
9.10	1099				
9.16	* 1092				
9.23	1083		1111–1030	233	65
9.31	* 1074	−2			
9.42	1062			123	
9.52	1050	4		135	
9.60	1042				
9.79	1021	9	1030–1009	60	2.1
9.98	1002		1009–987	50	2.4
10.21	979			5.6	
10.47	955	−4	939–841		50
10.89	918			128	
11.11	900	2		70	
11.35	881	9		86	
11.64	859	9		22	
11.88	* 842				
12.15	823		837–789	41	7.5
12.50	** 800				
12.89	776	−4	789–765	115	8.7
13.37	748			65	
			765–644		73
13.59	736			61	
14.03	713	−11		131	
14.12	* 708	7			
14.29	* 700				
14.53	688	−1	730–644	189	49
14.93	* 670				
15.17	* 659				
15.60	* 641	3			
15.95	* 627	4			
16.47	607	6	644–590	113	15
17.09	585		644–565	69	25
17.33	* 577	1			
17.92	* 558	2			
18.25	548	7	564–524	50	2.6
19.08	* 524	−3			
19.49	513			48	
19.88	503	−1		45	
21.05	* 475	−5			
21.98	455	−9			
22.83	438	−5		2.7	
23.36	428			4.4	
23.70	422	−1			
24.33	411	−7		4.2	

(*) New band at low T; (**) New band at high T; (***) from −180 °C to +50 °C.

**Table 3:** The [ε] and [ψ] parameters of CYTIDINE IR emissions.

Wavelength at 50 °C	Wavenumber at 50 °C	Band Shift Δ*ν_a_* −180 °C to +180 °C	Integration Region	[*ε*]	[*ψ*]
μm	cm^−1^	cm^−1^	μm	M^−1^ cm^−1^	km/mol
2.90	3448	−24	3800–2354	227	1106
2.99	3349	−1		266	
3.05	3278	5	3512–2978	225	444
3.09	3232	6		237	
3.12	* 3203				
3.24	3087	2		166	
3.37	2963	−2		113	
3.39	2952			123	
3.42	2920	1	2891–2801	131	41
3.45	2896	−3		115	
3.51	2850	−2		85	
3.67	* 2727				
5.10	1960	12	1788–1552	12	273
6.00	* 1667				
6.02	1660	7	1726–1620	330	80
6.05	* 1652	2			
6.08	1646	−8	1620–1559	354	55
6.24	1603	1		371	
6.33	1581	7		186	
6.51	* 1537				
6.53	1531	6	1552–1515	247	21
6.67	1500	11	1515–1442	360	55
6.85	1460			96	
6.98	1432	−2	1439–1419	90	2,3
7.10	* 1409	9			
7.12	1404			111	
7.27	1376	4	1419–1352	108	24
7.27	* 1375				
7.45	1343			55	
7.64	1309	1	1322–1230	90	41
7.75	1291	7		209	
7.79	* 1283				
7.90	* 1266				
8.01	1248	2		76	
8.24	1213	5	1230–1176	117	16
8.33	* 1200	11			
8.39	1192			71	
8.67	1153	9	1174–1001	91	152
8.80	1136	10		123	
8.97	* 1115				
9.08	1101	5	1124–1078	242	30
9.13	* 1095				
9.35	* 1070				
9.43	* 1061				
9.48	1055	3	1078–1001	295	54
9.66	1035	1		157	
10.16	984		998–957	107	13
10.60	943	4	957–896	104	12
11.48	871	3	885–863	53	3.9
11.71	854	4	863–832	63	5.5
11.90	840	5		25	
12.25	816	12	839–806	121	7.9
12.64	791	4	806–769	229	21
13.00	* 769				
13.23	756			49	
13.61	735	−9		62	
13.99	715	15	769–639	140	48
14.10	* 709				
15.11	662			49	
15.80	633			37	
16.08	622	5	639–607	101	6.7
16.69	599	2	607–579	96	4.2
17.39	* 575	10			
17.73	* 564	21			
18.02	555	17	579–480	103	23
21.32	** 469				
21.65	* 462	29			
22.99	435	−2	461–425	20	3.1
22.57	** 443				
23.75	421	−9			
24.10	415	−4			
24.63	406	−1			

(*) New band at low T; (**) New band at high T.

**Table 4:** The [*ε*] and [*ψ*] parameters of URIDINE IR emissions.

Wavelength at 50 °C	Wavenumber at 50 °C	Band Shift Δ*ν_a_* −180 °C to +180 °C	Integration Region	[*ε*]	[*ψ*]
μm	cm^−1^	cm^−1^	μm	M^−1^ cm^−1^	km/mol
2.93	* 3413				
2.99	3346			195	
3.02	* 3315				
3.21	3114		3714–2286	117	1030
3.22	3104	3	2460–2534	126	25
3.28	3051	−9		93	
3.37	2964			128	
3.42	2926	1		129	
3.47	2878	9		91	
3.57	2801	−1		99	
3.59	* 2785				
5.61	1781			56	
5.88	* 1701				
5.95	1681	−15	1977–1513	246	303
5.96	* 1677				
5.98	* 1673				
6.00	* 1668				
6.79	1472		1506–1447	102	17
6.85	* 1460	−3			
6.97	* 1435				
7.03	1422		1446–1410	97	5.7
7.16	1397	8	1410–1376	123	10
7.21	* 1387				
7.34	1363		1375–1335	75	6.5
7.41	1349			52	
7.45	* 1343				
7.58	1320	6	1334–1297	74	6.7
7.76	* 1289				
7.88	1269	4	1297–1225	181	42
7.94	* 1259				
8.26	1210	4	1225–1184	83	11
8.51	1175		1184–1161	28	1.7
8.68	* 1152				
8.72	* 1147				
8.80	1137		1161–1125	77	7.8
9.02	* 1109				
9.12	1097		1125–1065	203	40
9.30	1075		1125–993	137	97
9.49	1054	−1	1065–1038	173	10
9.68	1033			100	
9.93	1007			34	
10.18	982	3	993–962	63	6
10.19	* 981				
10.49	953	4	962–928	39	3.6
10.63	941			19	
10.80	* 926				
11.03	907		928–887	27	4.5
11.39	878	−17	887–861	29	2.3
11.57	864			14	
11.72	853	−3	861–840	33	1.9
12.03	831	8	840–816	73	5.1
12.41	806	−5	816–798	20	1.2
12.64	791			28	
12.90	775	3		66	
13.05	766	2	798–726	110	26
13.28	753			46	
13.97	716	6	726–701	50	5,1
14.81	* 675				
15.15	* 660				
15.53	* 644				
15.82	632	−1	650–619	61	2.1
16.47	607	−1	619–587	61	3.4
17.42	574	5	587–559	64	4.6
18.15	551	1	559–529	45	3.0
20.12	* 497				
18.76	** 533				
22.12	452		472–421	57	10
22.73	440			43	
23.75	** 421				
24.15	414		420–400	25	1.8

(*) New band at low T; (**) New band at high T.

## Data Availability

All laboratory data reported in this paper are fully available from the authors.
